# Oxidative Stress Impairs the Heat Stress Response and Delays Unfolded Protein Recovery

**DOI:** 10.1371/journal.pone.0007719

**Published:** 2009-11-11

**Authors:** Masaaki Adachi, Yaohua Liu, Kyoko Fujii, Stuart K. Calderwood, Akira Nakai, Kohzoh Imai, Yasuhisa Shinomura

**Affiliations:** 1 Division of Molecular Oncology and Molecular Diagnosis, Sapporo Medical University School of Medicine, Sapporo, Japan; 2 First Department of Internal Medicine, Sapporo Medical University School of Medicine, Sapporo, Japan; 3 Department of Neurosurgery, The First Clinical College of Harbin Medical University, Harbin, People's Republic of China; 4 Department of Radiation Oncology Beth Israel Deaconess Medical Center, Harvard Medical School, Boston, United States of America; 5 Department of Biochemistry and Molecular Biology, Yamaguchi University School of Medicine, Ube, Japan; Texas A&M University, United States of America

## Abstract

**Background:**

Environmental changes, air pollution and ozone depletion are increasing oxidative stress, and global warming threatens health by heat stress. We now face a high risk of simultaneous exposure to heat and oxidative stress. However, there have been few studies investigating their combined adverse effects on cell viability.

**Principal Findings:**

Pretreatment of hydrogen peroxide (H_2_O_2_) specifically and highly sensitized cells to heat stress, and enhanced loss of mitochondrial membrane potential. H_2_O_2_ exposure impaired the HSP40/HSP70 induction as heat shock response (HSR) and the unfolded protein recovery, and enhanced eIF2α phosphorylation and/or XBP1 splicing, land marks of ER stress. These H_2_O_2_-mediated effects mimicked enhanced heat sensitivity in HSF1 knockdown or knockout cells. Importantly, thermal preconditioning blocked H_2_O_2_–mediated inhibitory effects on refolding activity and rescued HSF1 +/+ MEFs, but neither blocked the effects nor rescued HSF1 -/- MEFs. These data strongly suggest that inhibition of HSR and refolding activity is crucial for H_2_O_2_–mediated enhanced heat sensitivity.

**Conclusions:**

H_2_O_2_ blocks HSR and refolding activity under heat stress, thereby leading to insufficient quality control and enhancing ER stress. These uncontrolled stress responses may enhance cell death. Our data thus highlight oxidative stress as a crucial factor affecting heat tolerance.

## Introduction

Exposure to excess reactive oxygen species (ROS) induces oxidative stress, which is believed to be associated with various human pathologies, including aging, carcinogenesis, and neurodegenerative disorders [1], [2]. These diseases may be developed from accumulation of oxidized cellular components, e.g., DNAs, proteins and lipids. Although these oxidized components are quickly repaired or eliminated, oxidation may alter their functional effects, thereby impairing various cellular processes. To understand the roles of oxidization in these pathologies, it is crucial to clarify which functions change under oxidative stress.

Heat shock response (HSR) induces numerous heat shock proteins (HSPs), many of which are chaperone proteins that assist in protein folding and protect cellular homeostasis against heat and other stress stimuli [3], [4]. Under heat stress conditions, heat shock transcription factor 1 (HSF1) binds to a DNA sequence motif, the heat shock element (HSE), and activates transcription of genes encoding many chaperone proteins, including the hsp70 and hsp40 genes. HSF1 plays a crucial role in this process, since HSF1 knockout impairs HSR and enhances sensitivity to heat [5], [6]. Thus, induction of chaperone molecules obviously protects cells from heat-induced cell death.

Global warming, air pollution and destruction of the ozone layer threaten human health. Temperatures are gradually increasing, while destruction of the ozone layer raises levels of solar ultraviolet (UV) radiation. Considering that air pollution and UV radiation induce cellular ROS accumulation [7], we are exposed to a double risk from heat and oxidative stress simultaneously. In this study, we investigated a possible linkage between heat and oxidative stress, and found that oxidative stress strongly enhanced heat sensitivity. Importantly, H_2_O_2_ clearly inhibited the upregulation of HSP70/HSP40 transcription under heat stress and blocked the protein refolding ability. Since H_2_O_2_ enhanced or prolonged heat-induced eIF2α phosphorylation and XBP1 splicing, inhibition of HSR may cause denatured proteins to accumulate and enhance heat sensitivity. We here present the effects of HSR inhibition under oxidative stress and suggest oxidative stress as a pivotal factor affecting heat tolerance.

## Results

### Enhancing Effects of H_2_O_2_ on Heat Induced Cell Death

**Figure 1:**
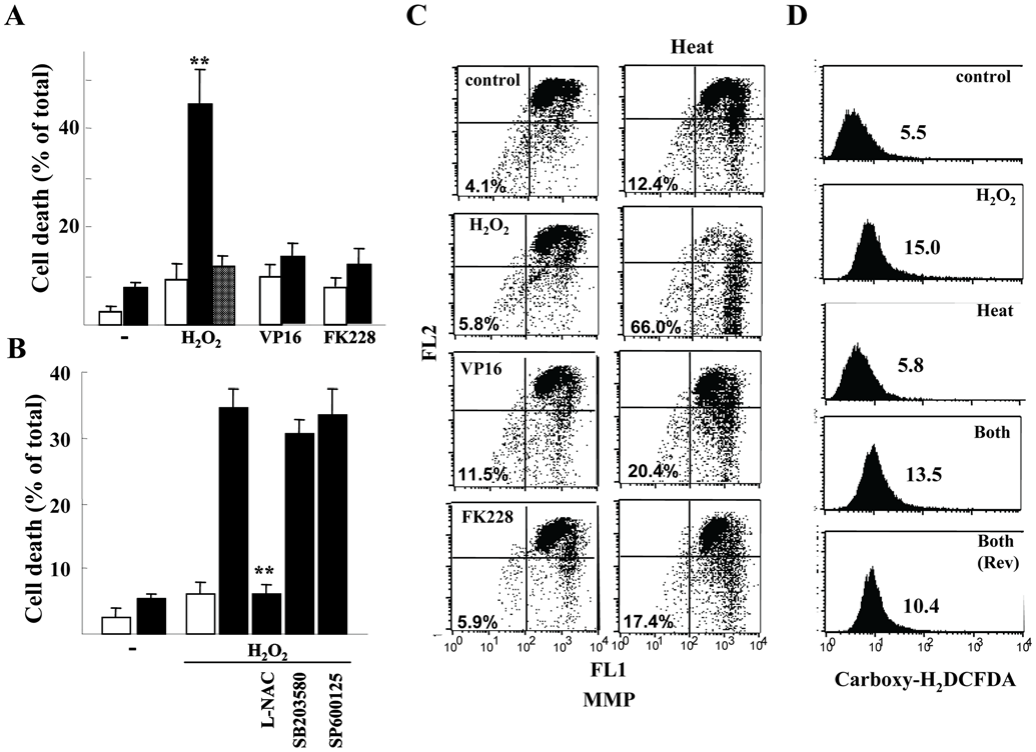
Cell death in H_2_O_2_ and/or heat-treated T98G cells. *A*) Trypan blue exclusion assay. Cell death 24 h after heat exposure (44°c for 20 min) (closed bars) with the indicated pretreatments (0.25 mM H_2_O_2_, 25 µg/ml VP16 or 50 nM FK228). Alternatively, cells were exposed to heat and thereafter treated with 0.25 mM H_2_O_2_ as reverse treatment (gray bar) or unexposed to heat (open bars). **, *P*<0.01 compared with cells treated alone as indicated. *B*) Cell death was evaluated 20 h after heat exposure. Cells were pretreated with 5 mM L-NAC, 10mM SB203580 or 10 mM SP600125 for 2 h, thereafter treated with 0.25 mM H_2_O_2_, and unexposed (open) or exposed (closed bars) to 44°c for 20 min. **, *P*<0.01 compared with heat/H_2_O_2_-treated cells. In *A* and *B*, error bars indicate the mean ± S.D. of data from three separate experiments. *C*) Disruption of Δψm. Cells were cultured for 20 h after the indicated treatments as described in *A*), and then intracellular DePsipher fluorescence was detected. Numbers indicate % of cells showing loss of Δψm. *D*) ROS generation. Carboxy-H_2_DCFDA fluorescent signals 2 h after treatment with 0.25 mM H_2_O_2_ (H_2_O_2_), heat exposure (44°c for 20 min; Heat) or both (Both). Both (Rev) indicates reverse treatment (heat exposure before H_2_O_2_ treatment). In representative histograms, numbers indicate mean fluorescence intensity.

We first investigated the effects of H_2_O_2_ on the heat sensitivity of human malignant glioma T98G cells. Treatment with 0.25 mM H_2_O_2_ prior to heat (44°c) exposure for 20 min strongly increased cell death (approximately 45%); however, H_2_O_2_ alone did not cause any distinct toxic effect (Figure 1A). Pretreatment with the free radical scavenger L-N-acetylcystein (L-NAC) almost completely blocked H_2_O_2_-mediated enhanced cell death. In contrast, two strong anticancer agents VP16 (a topoisomerase II inhibitor) and FK228 (an HDAC inhibitor), having no distinct ROS generation, had no significant effect on cell viability. Furthermore, neither the p38 kinase inhibitor SB203580 nor the JNK inhibitor SP600125 affected viability (Figure 1B). These data suggest that H_2_O_2_–mediated oxidative stress specifically sensitized T98G cells to heat, and stress kinases may be only marginally involved in the enhancing effect. The H_2_O_2_–mediated enhanced sensitivity was also exhibited by an increased loss of mitochondrial membrane potential (MMP) (Figure 1C).

To determine the molecular mechanism for the stress, we monitored ROS generation after heat exposure with or without H_2_O_2_ pretreatment. H_2_O_2_ clearly increased ROS generation, whereas heat exposure alone did not, and its combination with H_2_O_2_ (including reverse treatment) exhibited no enhancing effect (Figure 1D), thereby excluding the possibility that heat stress and oxidative stress synergistically augment ROS generation.

### Inhibitory Effects of H_2_O_2_ on HSR

**Figure 2:**
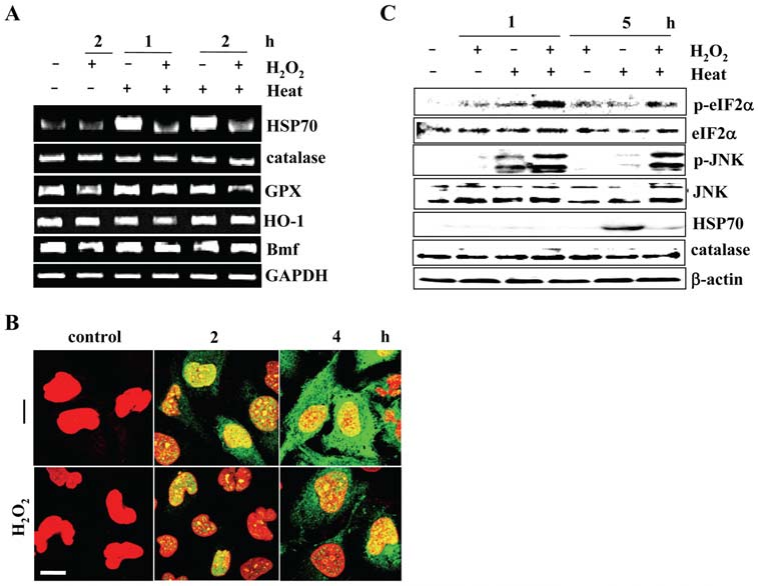
Effects of H_2_O_2_ on HSR and expression of scavenger enzymes. *A*) Total RNA was harvested from T98G cells at the indicated hours after the indicated treatments (44°c for 20 min and/or 0.25 mM H_2_O_2_) and transcripts of the indicated genes were evaluated by RT-PCR. *B*) Confocal microscopical detection of HSP70. Cells were pretreated with 0.25 mM H_2_O_2_ (H_2_O_2_) or untreated (control) and fixed at 2 or 4 h after heat exposure. HSP70 expression was immuunohistochemically detected using the anti-HSP70 antibody and cells were counter-stained with propidium iodide (PI). Bar indicates 10 µm. *C*) eIF2α and JNK phosphorylation. At 1 and 5 h after indicated treatments as described above, eIF2α and JNK activities were evaluated by western blots using anti-phosphorylated eIF2α and anti-phosphorylated JNK antibodies, respectively. HSP70 and catalase expression levels were also evaluated. Anti-β-actin, anti-eIF2α and anti-JNK protein antibodies show equal loading of protein samples.

We next investigated the effects of H_2_O_2_ on HSR. When cells were exposed to heat (44°c for 20 min), HSP70 mRNA was rapidly induced, but H_2_O_2_ pretreatment clearly inhibited the induction of HSP70 mRNA and its products (Figure 2A-C). In contrast, H_2_O_2_ pretreatment had no effect on the other mRNA expression levels, i.e., catalase, glutathion peroxidase (GPX), heme oxygenase-1 (HO-1), and Bmf mRNA (Figure 2A). It was noted that H_2_O_2_ strongly increased and prolonged phosphorylation levels of eIF2α or JNK (Figure 2C).

**Figure 3:**
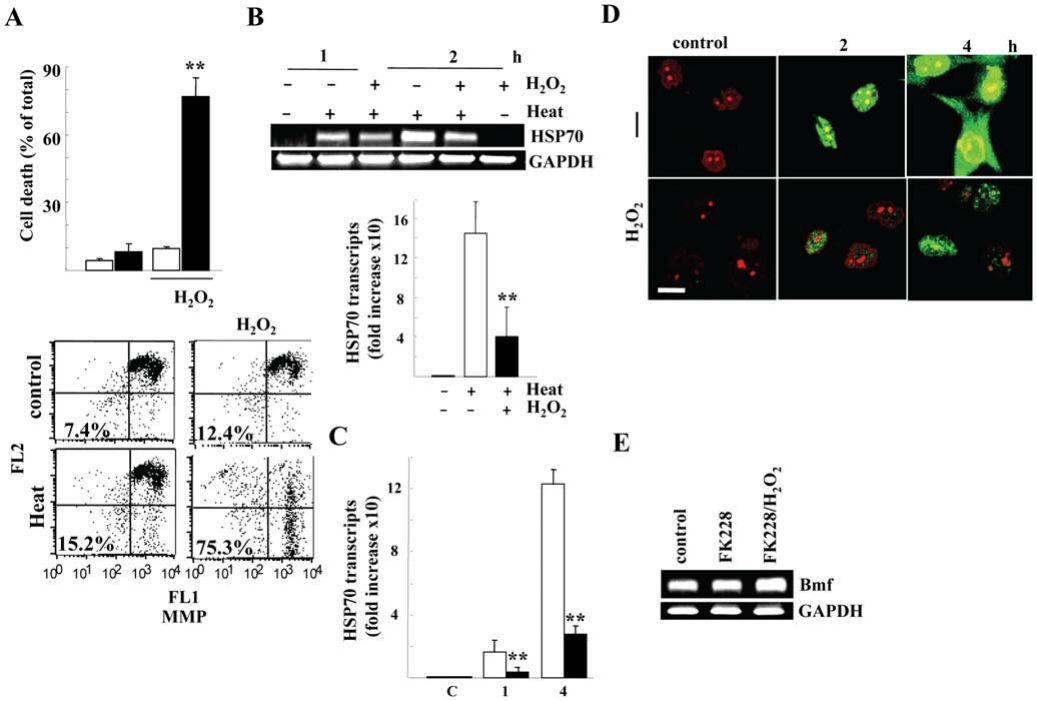
Effect of H_2_O_2_ on heat-exposed A172 cells. *A*) Cell death at 24 h (upper) and disruption of Δψm (lower panel) at 20 h after heat (44°c for 20 min) exposure (closed bars) with or without 0.25 mM H_2_O_2_. Numbers indicate % of cells showing loss of Δψm. *B*) HSP70 transcripts. Total RNA was harvested from cells treated with the indicated and transcripts of the indicated genes were evaluated by RT-PCR (upper). HSP70 transcripts were determined by real-time PCR and normalized to GAPDH levels (lower panel). *C*) Time-course of HSP70 transcripts. At the indicated hours after heat exposure with (closed) or without (open bars) 0.25 mM H_2_O_2_, HSP70 transcripts were determined by real-time PCR and normalized to GAPDH levels In *A*-*C*, error bars indicate the mean ± S.D. of data from three separate experiments and **, *P*<0.01 compared with H_2_O_2_-untreated cells. *D*) Confocal microscopical detection of HSP70. Cells were pretreated with (H_2_O_2_) or without (control) 0.25 mM H_2_O_2_ and fixed at the indicated hours after heat (44°c for 20 min) exposure. HSP70 expression was immunohistochemically detected using the anti-HSP70 antibody counter-stained with PI. Bar indicates 10 µm. *E*) Bmf transcripts evaluated by RT-PCR of total RNA harvested at 6 h after treatment with the indicated agents for 6 h In *B* and *E*, GAPDH mRNA levels ensure that the RNA was correctly quantified.

In another glioma A172 cell line, H_2_O_2_ similarly enhanced heat-induced cell death and loss of MMP (Figure 3A), and inhibited induction of HSP70 mRNA (Figure 3B and 3C) and its products (Figure 3D). Consistent with the previous data showing a marginal effect of p53 status on heat sensitivity [8], there was no big difference in heat sensitivity and effect of H_2_O_2_ between T98G (carrying a mutant p53) and A172 (carrying a wild-type p53) cells.

### H_2_O_2_ Targets HSF1-Mediated Transcription, but Not through Inhibition of HSF1 Binding Ability

**Figure 4:**
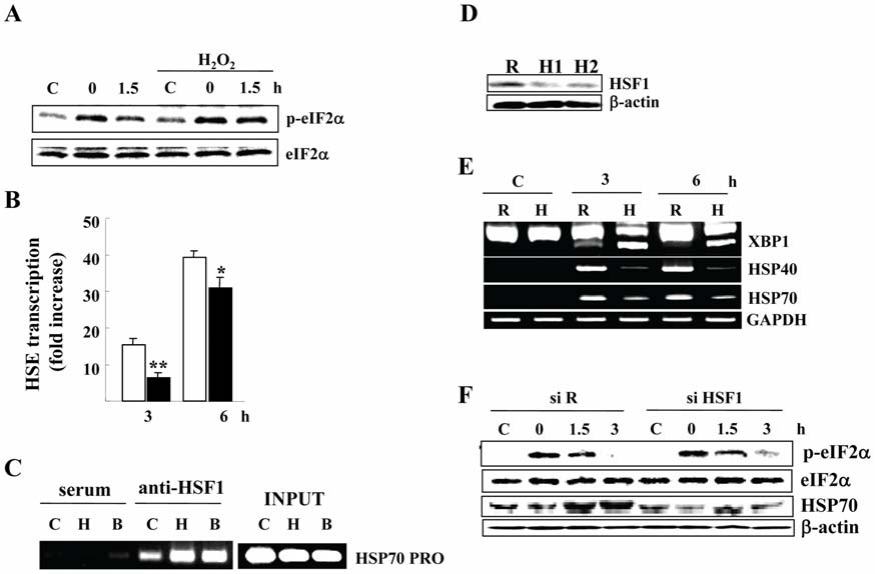
ChIP assay and knockdown of HSF1 transcripts. *A*) eIF2α phosphorylation in A172 cells. At the indicated hours after heat exposure (44°c for 20 min) with or without 0.25 mM H_2_O_2_ pretreatment, eIF2α activity was evaluated by western blots, and anti-eIF2α protein antibody shows equal loading of protein samples. *B*) Effect of H_2_O_2_ pretreatment on HSE transcription. A172 cells were transiently co-transfected with the pHSE and the pRL-TK reporter genes, pretreated with 0.25 mM H_2_O_2_ (closed bars), incubated for the indicated hours after heat exposure (43°c for 20 min), and their transcription activity was evaluated by dual luciferase assays. Bars display the mean ± S.D. of data from three separate experiments and *, *P*<0.05, **, *P*<0.01 compared with H_2_O_2_-unexposed cells. *C*) Chromatin immunoprecipitation assays. Cells (C; control) were incubated 2 h after heat exposure (44°c for 20 min) with (B; both) or without (H; heat alone) 0.25 mM H_2_O_2_ pretreatment. They were then fixed with formaldehyde, and immunoprecipitated with antibodies against HSF1 (anti-HSF1) or nonimmunized rabbit serum (serum). Immunoprecipitates were amplified by HSF1 promoter primers. A fixed portion of the total input was also examined by PCR (INPUT). *D*) HSF1 knockdown. Total cell lysates were harvested 36 h after transfection with siRandom (R) or two different siHSF1 (H1 and H2). *E*) XBP1 splicing. Total RNA was prepared from cells transfected with siRandom (R) or siHSF1 (H; H1). After transfection, cells were exposed to heat (44°c for 20 min) and incubated for the indicated hours. The indicated transcripts were evaluated by RT-PCR. GAPDH mRNA levels ensure that the RNA was correctly quantified. *F*) eIF2α phosphorylation. After exposure to heat (44.5°c for 20 min), eIF2α phosphorylation and HSP70 expression were evaluated by western blots. Anti-eIF2α protein antibody (in *A* and *F*) and anti-β-actin antibody (in *D* and *F*) show equal loading of protein samples.

H_2_O_2_ induced prolonged eIF2α phosphorylation at 1.5 h after heat exposure (Figure 4A), suggesting that H_2_O_2_ prolongs translational block. We next employed luciferase reporter assays using the expression vector carrying HSE in A172 cells. Heat exposure clearly elevated HSE-mediated transcription and subsequent translation, but H_2_O_2_ significantly decreased their activation (Figure 4B), confirming the inhibitory effect of H_2_O_2_ on HSR.

As described above, HSF1-mediated HSR is a central pathway to induce chaperone molecules and to protect cells from heat-induced cell death. Chromatin immunoprecipitation (ChIP) assay however showed that H_2_O_2_ barely affected the binding ability of HSF1 to HSE upstream of HSP70 gene (Figure 4C). Comparable levels of HSE DNA fragments were also recovered in immunoprecipitates either heat alone or heat exposure to H_2_O_2_–treated cells in another cell line (data not shown), indicating that H_2_O_2_ did not inhibit HSF1 binding ability. Although molecular mechanism(s) for the H_2_O_2_-mediated HSR inhibitory effect remains unclear, H_2_O_2_ treatment rather enhanced the FK228-mediated Bmf mRNA induction (Figure 3E), suggesting that H_2_O_2_ does not inhibit transcription activity in general.

We next employed transfection of siRNA specifically targeting to HSF1 mRNA in A172 cells. Knockdown of HSF1 mRNA clearly decreased HSF1 expression (Figure 4D), inhibited HSP70/40 mRNA induction and enhanced/prolonged XBP1 splicing after heat exposure (Figure 4E). In addition, HSF1 knockdown inhibited HSP70 protein synthesis and induced prolonged phosphorylation of eIF2α (Figure 4F), an effect similar to that of H_2_O_2_. These data support that an inhibition of HSF1-mediated transcription is crucial for H_2_O_2_–mediated enhanced heat sensitivity.

### Inhibitory Effect of H_2_O_2_ on Protein Refolding Activity

**Figure 5:**
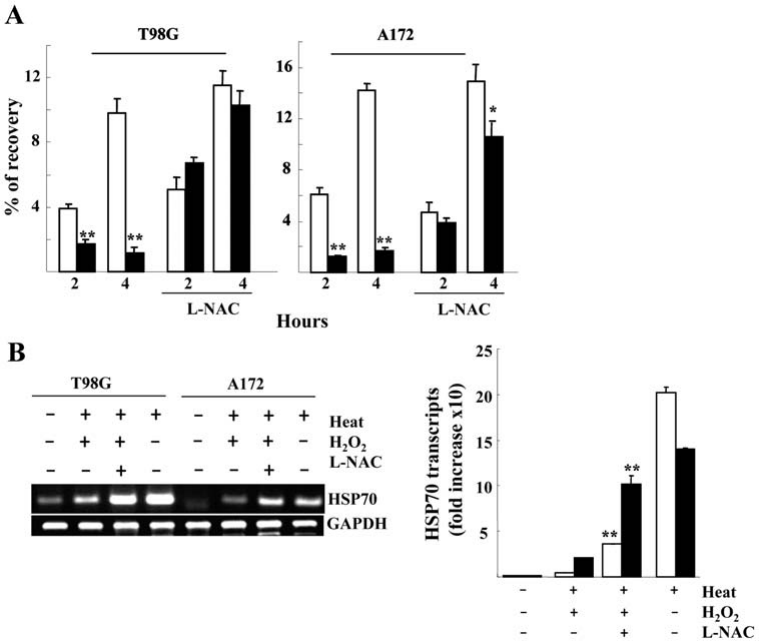
Recovery of protein folding activity. *A*) Refolding activity. Cells were transiently co-transfected with the pGRE/RL-TK reporter genes, treated with 5 mM dexamethazone for 10-12 h, exposed to heat (44°c for 20 min) with (closed) or without (open bars) 0.25 mM H_2_O_2_ pretreatment, incubated for 2 to 4 h and luciferase activity was measured. Cells were also incubated with 5 mM L-NAC for 30 min prior to 0.25 mM H_2_O_2_ pretreatment. *, *P*<0.05, **, *P*<0.01 compared with H_2_O_2_-untreated cells. *B*) HSP70 transcripts. Total RNA was harvested from cells treated as indicated and HSP70 transcripts were evaluated by RT-PCR (left). HSP70 transcripts were determined by real-time PCR and normalized to GAPDH levels (right panel). **, *P*<0.01 compared with heat/H_2_O_2_-treated cells. Columns display the mean ± S.D. of data from three separate experiments.

HSF1 knockdown or H_2_O_2_–mediated HSR interference similarly augmented or prolonged eIFα phosphorylation, a landmark of ER stress [9]. These effects suggest that after heat exposure, unfolded proteins may accumulate more in H_2_O_2_-pretreated cells. We thus monitored protein refolding activity with or without H_2_O_2_. H_2_O_2_ pretreatment clearly inhibited refolding activity in both T98G and A172 cells and the inhibition was mostly blocked by pretreatment with L-NAC (Figure 5A). The unfolded protein recovery corresponded well with the recovery of HSP70 mRNA induction (Figure 5B), suggesting that H_2_O_2_–mediated oxidative stress inhibits HSR and subsequent refolding activity.

### H_2_O_2_ Pretreatment Sensitizes HSF1 +/+ MEFs at a Similar Level of HSF1 -/- MEFs

**Figure 6:**
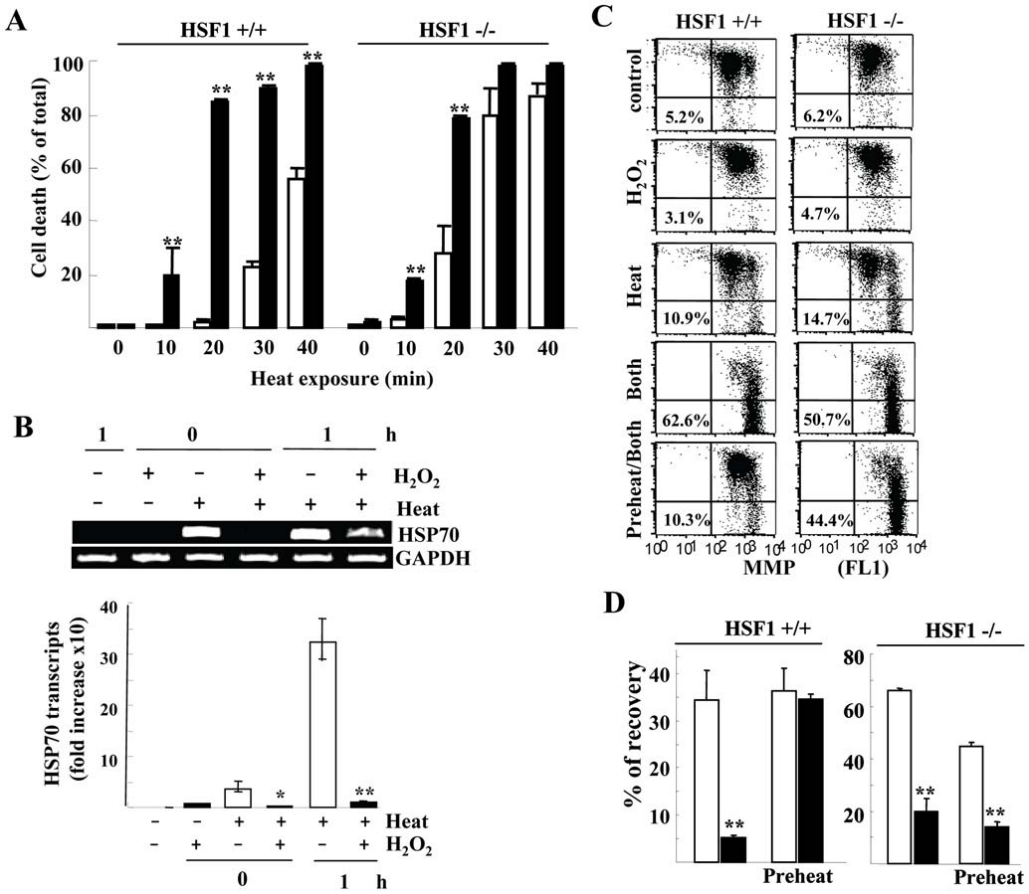
Effect of H_2_O_2_ on heat-exposed HSF +/+ and -/- MEFs. *A*) Cell death at 24 h after heat exposure (42.5°c for the indicated min) with (closed) or without (open bars) 0.5 mM H_2_O_2_ pretreatment. *B*) HSP70 transcripts evaluated by RT-PCR transcription at the indicated hours after the indicated treatments (upper) were determined by real-time PCR with normalization to GAPDH levels (lower panel). *C*) Preconditioning effect on disruption of Δψm. Both MEF cells were treated as indicated, heat (Heat; 42.5°c for 20 min), 0.5 mM H_2_O_2_ treatment (H_2_O_2_) and both treatments (Both). As thermal preconditioning, cells were preheated (40.5°c for 30 min 10 h) prior to both treatments (Preheat/Both). Cells were cultured for 20 h after heat exposure and incubated with DePsipher solution. Numbers indicate % of cells showing loss of Δψm. *D*) Effect of H_2_O_2_ pretreatment on refolding activity. Refolding activity was evaluated by recovery of luciferase activity 5 h after heat exposure (42.5°c for 20 min) with (closed) or without (open bars) 0.5 mM H_2_O_2_ pretreatment. Cells were also treated by thermal preconditioning (Preheat) as described in *C*. In *A*, *B* and *D*, columns display the mean ± S.D. of data from three separate experiments and *, *P*<0.05; **, *P*<0.01 compared with H_2_O_2_-untreated cells.

To further explore the correlation between HSR and refolding activity, we used HSF1 -/- and +/+ murine embryonal fibroblasts (MEF). As previously described, HSF1 -/- MEF cells were highly sensitive to heat [10], and approximately 30% of cells lost viability by heat exposure alone (42.5°c for 20 min), while the same treatment barely induced cell death in wild-type MEF cells (Figure 6A). Interestingly, H_2_O_2_ pretreatment highly sensitized both MEFs to heat, the cells becoming equally sensitive (85% vs 80%). Indeed, H_2_O_2_ pretreatment strongly inhibited HSP70 mRNA induction in HSF1 +/+ MEFs (Figure 6B) and increased loss of MMP at a higher level than in HSF1 -/- MEFs (62.6% vs 50.7%) (Figure 6C).

### Lack of Thermal Preconditioning Effects in HSF1 -/- MEFs

**Figure 7:**
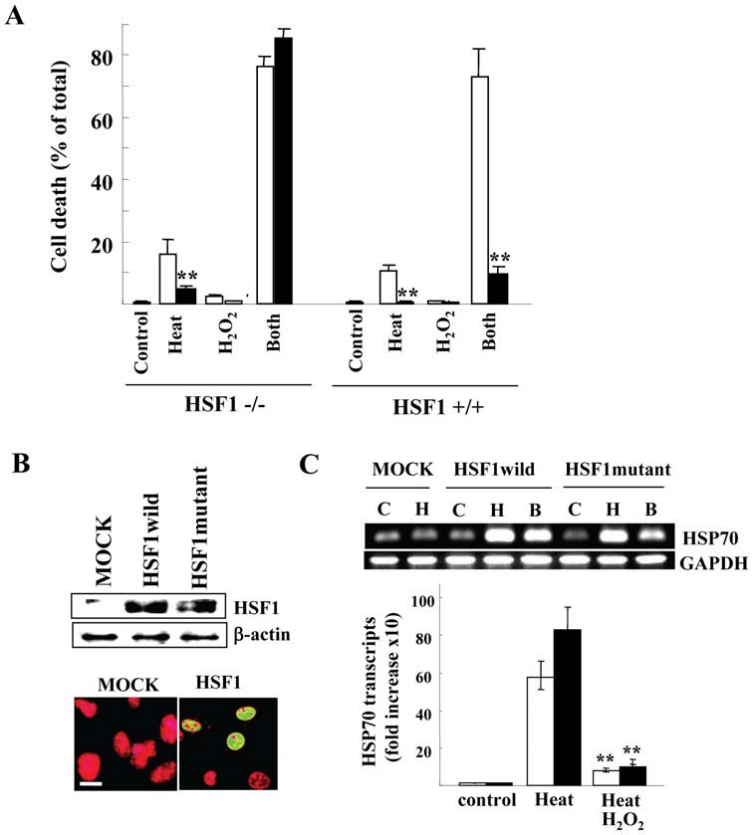
Effect of thermal preconditioning on H_2_O_2_-treated HSF +/+ and -/- MEFs. *A*) Cell death at 24 h after indicated treatments with (closed) or without (open bars) thermal preconditioning. Both MEFs were treated as indicated, heat (Heat; 42.5°c for 20 min), 0.5 mM H_2_O_2_ treatment (H_2_O_2_) and both treatments (Both). **, *P*<0.01 compared with un-preconditioned cells. *B*) HSF1 overexpression. The indicated HSF1 expression vectors were transfected into HSF1-/- MEFs and cell lysates were harvested at 2 days after transfection. β-actin demonstrates equal loading of protein samples (upper). Nuclear localization of HSF1 in HSF1-rescued MEFs was evaluated by confocal microscopy. The nucleus was counterstained with PI and bar indicates 10 µm. (lower panel). *C*) HSP70 transcripts were evaluated by RT-PCR transcription in cells 1 h after exposure to heat alone (H; 42.5°c 20 min), heat after 0.5 mM H_2_O_2_ pretreatment (B) or untreated cells (C). GAPDH mRNA levels ensure that the RNA was correctly quantified (upper). After indicated treatments, HSP70 transcripts in transfectants expressing wild-type (open) or mutant HSF1 (closed bars) were determined by real-time PCR and normalized to GAPDH levels (lower panel). **, *P*<0.01 compared with H_2_O_2_-untreated cells. In *A* and *C*, error bars indicate the mean ± S.D. of data from three separate experiments and

We next investigated thermal preconditioning, which largely prevents heat stress, i.e., cells pretreated by mild heat become heat-resistant [11]. Indeed, cell death (Figure 7A) and loss of MMP (Figure 6C) were strongly inhibited by preconditioning in wild-type MEF cells, but this effect was barely observed in HSF1 -/- MEFs (cell death; 9% vs 86%, loss of MMP; 10.3% vs 44.4%). As observed in T98G and A172, refolding activity was substantially inhibited by H_2_O_2_ pretreatment in both MEF cells, but the inhibition was more in HSF1 +/+ MEFs (Figure 6D). Preconditioning mostly reversed the H_2_O_2_–mediated decreased refolding activity only in HSF1 +/+ MEFs (Figure 6D), indicating that thermal preconditioning requires HSF1-mediated signals, and is able to cancel H_2_O_2_ actions. These data suggest that the biological effects of H_2_O_2_ mostly arise from HSF1-mediated HSR inhibition, and a tight linkage between unfolded protein recovery and protection of MMP disruption.

### H_2_O_2_–Mediated HSF1 Modification Is Unlikely

A previous report shows that the proinflammatory protein kinase MAPKAP kinase 2 (MK2) directly phosphorylates HSF1 at serine 121 and inhibits activity by decreasing its ability to bind the HSE [12]. Phosphorylation of HSF1 might explain H_2_O_2_–mediated inhibition of HSR. Expression vectors of a wild-type or a mutant HSF1 carrying S121A were transiently transfected into HSF1 -/- MEFs, and comparable levels of HSF1s were expressed in the nucleus (the transfection efficiency was approximately 50%) (Figure 7B). Heat exposure clearly induced HSP70 mRNA and H_2_O_2_ pretreatment inhibited the induction similarly in both transfectants (Figure 7C), suggesting that HSF1 phosphorylation is marginal for the mechanism of H_2_O_2_-mediated HSR inhibition. Indeed, we could not detect any significant phosphorylation of HSF1 by H_2_O_2_ treatment using the antibody specifically to detect phosphorylated HSF1 at Ser121 (data not shown), and this is consistent with our observation showing no inhibitory effect of stress kinase inhibitors (Figure 1B).

### HSF1 Overexpressed Actions Are Cancelled by H_2_O_2_–Treatment

**Figure 8:**
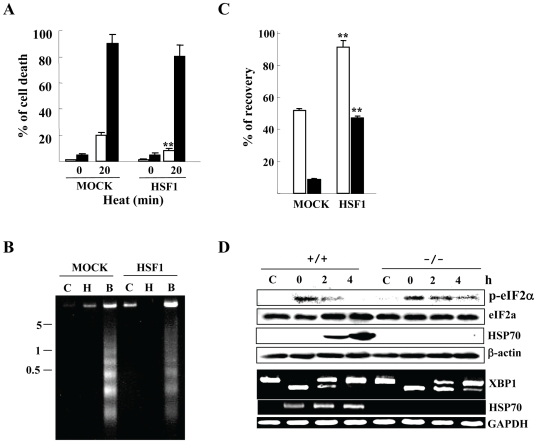
HSF1 rescue into HSF -/- MEFs. *A*) Cell death at 24 h after heat exposure (42.5°c) for 20 min with (closed) or without (open bars) 0.5 mM H_2_O_2_ pretreatment. **, *P*<0.01 compared with MOCK transfectants. *B*) DNA fragmentation. Small molecular DNAs were prepared from cells (C: control, H: 42.5°c for 10 min, B: heat and 0.5 mM H_2_O_2_) 24 h after heat exposure. Numbers indicate molecular weights (kilo bases). *C*) Effect of H_2_O_2_ pretreatment on refolding activity. Cells were transiently co-transfected with the wild-type pHSF1 expression vector (HSF1) or its vehicle (MOCK) with the pGRE/RL-TK reporter genes, treated with 5 mM dexamethazone for 10-12 h, treated by thermal preconditioning as described in Fig. 6*D*, exposed to heat (42.5°c for 20 min) with (closed) or without (open bars) 0.5 mM H_2_O_2_ pretreatment, incubated for 5 h and luciferase activity was measured. **, *P*<0.01 compared with MOCK transfectants. *D*) eIF2α phosphorylation and XBP1 splicing. After exposure to heat (43.5°c for 20 min), HSF +/+ and -/- MEFs were harvested at the indicated hours. The eIF2α phosphorylation and HSP70 expression were evaluated by western blots using the indicated antibodies. Anti-β-actin antibody shows equal loading of protein samples. XBP1 splicing and HSP70 mRNA expression were evaluated by RT-PCR. GAPDH mRNA levels ensure that the RNA was correctly quantified. In *A* and *C*, error bars indicate the mean ± S.D. of data from three separate experiments.

We next investigated the effect of HSF1 overexpression on cell viability in HSF1 -/- MEFs. HSF1 overexpression actually decreased cell death induced by heat alone, but its protective effect was limited in H_2_O_2_–exposed cells (Figure 8A) and comparable levels of DNA fragmentation were detected in both transfectants exposed to H_2_O_2_ (Figure 8B). Thus, H_2_O_2_ treatment appeared to cancel HSF1-mediated protection against heat stress. As described above, preconditioning almost fully restored unfolded protein recovery in H_2_O_2_-treated HSF1 +/+ MEFs, but not in H_2_O_2_-treated HSF1 -/- MEFs (Figure 6D). The HSF1-rescued HSF1 -/- MEFs strongly restored the preconditioning effect even in H_2_O_2_–treated cells (Figure 8C). Considering transfection efficiency was nearly 50%, this recovery suggests that HSF1 is crucial for the preconditioning effect on refolding activity. In addition, cancellation of H_2_O_2_ action in HSR-completed (preconditioned) cells implicates that HSF1-mediated HSR is a primary target of H_2_O_2_.

### HSF1 -/- MEFs Exhibit More ER Stress After Heat Exposure

We finally monitored ER stress markers in both MEFs after heat exposure (43.5°c for 20 min). HSF1 -/- MEFs exhibited prolonged eIF2a phosphorylation and XBP1 splicing (Figure 8D), indicating that HSF1-mediated HSR plays a preventive role against heat-induced ER stress.

## Discussion

We here show that oxidative stress influences the HSR and the unfolded protein recovery, and decreases their protective functions against heat stress. Several reports link oxidative stress with heat stress and suggest synergistic augmentation of cell death, and report increased ROS generation in heat exposed cells [13]-[15]. However we neither detected increased ROS generation in heated cells nor in heated cells with H_2_O_2_. It is thus unlikely that their synergistic effects are primarily caused by enhanced ROS generation.

Oxidative stress neither decreased steady-state levels of all transcripts examined nor FK228-mediated Bmf mRNA induction. Thus, H_2_O_2_ appeared to specifically inhibit the induction of HSP70 and HSP40 mRNA in heat exposed cells. However, chromatin immunoprecipitation assay and mutation analysis suggested that H_2_O_2_ inhibition of HSR is mostly independent of HSF1 functions. Although we did not explore the precise molecular mechanism for the inhibitory effect of H_2_O_2_ on HSR, a previous report clearly indicates that H_2_O_2_ treatment elicits phosphorylation and ubiquitination of RNA polymerase II (RNAPII), leading to rapid, global, but transient repression of transcription [16]. Actually, we confirmed that H_2_O_2_ inhibition of HSR was restored 4 h after heat exposure (data not shown). This quick recovery may explain why H_2_O_2_ failed to inhibit FK228-mediated Bmf mRNA induction, making it unlikely that H_2_O_2_ affects steady-state levels of transcription.

Importantly, the short action may be sufficient to interfere with the HSR. Although HSR transcription is restored 4 h after heat exposure, HSR-linked chaperone proteins cannot be synthesized by translational block due to enhanced eIF2α phosphorylation. Although heat stress itself can induce eIF2α phosphorylation via the haem-regulated inhibitor (HRI) kinase [17], evoked ER stress also induces eIF2α phosphorylation via PERK [18]. Indeed, we clearly showed enhanced or prolonged eIF2α phosphorylation and/or XBP1 splicing [19] after heat exposure in H_2_O_2_-treated cells. They actually expressed much less HSP70 protein compared with H_2_O_2_-untreated cells. Thus, HSR may be a primarily sensitive signal affected by H_2_O_2_.

Several investigators have reported that HSP70 and other heat-inducible chaperones can reduce oxidative stress [20], and therefore it is possible that impaired HSR may further enhance ROS and contribute to augmented cell death especially when cells are exposed to persistent oxidative stress. In addition, we and others suggest a tight linkage between oxidative stress and ER stress [21], [22]. Indeed, we here demonstrated that oxidative stress impaired the HSR and enhanced or prolonged ER stress signals under heat stress. Collectively, these data implicate multiple stress signals, namely, oxidative stress, heat stress and ER stress, which develop in cells after heat exposure under oxidative stress and augment cell death.

Although heat induces a variety of illnesses including heat cramps, syncope, exhaustion and heat stroke [23], [24], there is little information about factors affecting heat stress-induced cell damage. Our data suggest that oxidative stress may be a crucial adverse factor increasing severity of these illnesses. Further *in vitro* and *in vivo* studies should clarify this possibility, which would make antioxidants promising drugs to prevent heat-induced illness. We are only on the threshold of this field. Beyond question, further studies to define anti-HSR functions in oxidative stress are essential.

## Materials and Methods

### Cell Culture

Human glioma cell lines, T98G and A172, obtained from the Japanese Cancer Research Resources Bank (Tokyo, Japan), were grown in d-MEM (SIGMA) supplemented with 10% fetal calf serum (FCS) and essential amino acids. HSF1 wild-type and knockout mouse embryonic fibroblasts (MEF) were described previously and were maintained in d-MEM with 10% FCS. To evaluate viability, cells were mixed with the same volume of 0.4% trypan blue solution, and immediately examined under light microscopy to determine whether they excluded the dye.

### Reagents and Antibodies

L-N-acetylcystein (L-NAC), etoposide, H_2_O_2_ and anti-β-actin antibody were supplied by Sigma (St. Louis, MO). The HDAC inhibitor bicyclic depsipeptide (FK228), which induces Bmf mRNA strongly [25], was kindly provided by Fujisawa Pharmaceutical Co. (Osaka, Japan). The anti-phospho-eIF2α was purchased from Cell Signaling Technology (Beverly, MA). The anti-JNK, anti-eIF2α, anti-HSP40 and anti-HSP70 antibodies were from Santa Cruz Biotechnology Inc. (Santa Cruz, CA). The anti-phospho-JNK antibody was from Promega CO (Madison, WI). The anti-HSF1 antibody was from Strassgen (Ann Arbor, MI).

### ROS Detection

Following treatment, cells were incubated with 10 µM 5-(and-6)-carboxy-2′,7′- dichlorodihydrofluorescein diacetate (carboxy-H_2_DCFDA) C-400 (Molecular Probes; Eugene, OR) for 30 min, after which they were washed, treated with the indicator and further incubated with complete medium for 2 h. ROS generation was determined using FACScan flow cytometer using CellQuest Software™, and fluorescent signals were displayed as histograms.

### Detection of Mitochodrial Membrane Potential (Δψm)

Following treatment, cells were incubated with DePsipher solution (Trevigen, Gaithersburg, MD) for 20 min, after which they were washed with PBS, resuspended with reaction buffer, Δψm was immediately determined using a FACScan flow cytometer (Becton Dickinson, Mountain View, CA). DePsipher is a lipophilic cation, which aggregates upon membrane polarization and forms an orange-red fluorescent compound. MMP disruption blocks aggregation of DePsipher, which reverts to its green monomeric green fluorescent form. Thus a decrease of the fluorescent signals (FL2) indicates loss of MMP.

### Western Blotting

After washing with ice-cold PBS, cells were lyzed by adding 200 µl of RIPA buffer (100 mM NaCl, 2 mM EDTA, 1 mM PMSF, 1% NP-40 and 50 mM Tris-HCl [pH 7.2]). Total cell lysates were collected and their protein concentration was evaluated using a Protein Assay (BioRad, Melville, NY). The lysates (20 µg/lane) were separated by 10 to 15% SDS-PAGE gels and then transferred to PVDF membranes (Millipore, Bedford, MA) at 20 V for 50 min. Membranes were soaked in 5% bovine serum albumin (BSA, Sigma) overnight. The membranes were incubated with primary antibodies overnight at 4°C, and thereafter incubated with the corresponding peroxidase-linked secondary antibodies (Amersham or MBL) for 1 h at room temperature. Signals were developed by a standard enhanced chemiluminescence (ECL) method following the manufacturer's protocol (Amersham).

### Reverse Transcriptase-PCR (RT-PCR) and Transfection

Total RNA was extracted with TRIzol (BRL Life and Technologies, MD). The indicated cDNAs were amplified from 1 µg of total RNA using High Capacity cDNA Reverse Transcription Kit (Applied Biosystems) with random primers. The cDNA products were analyzed on 2% agarose gel and confirmed by nucleotide sequencing. The following primer pairs were used for RT-PCR: HSP70 (human): 5′-acaagtgtcaagaggtcatctc-3′ and 5′-ctaatctacctcctcaatggtg-3′; HSP70 (mouse): 5′-acaagtgccaggaggtcatctc-3′ and 5′-tctaatccacctcctcgatggtg-3′; HSP40: 5′-caccatgggtaaagactactaccagac-3′ and 5′-tattggaagaacctgctcaagtacggttc-3′; XBP1: 5′-ccttgtagttgagaaccagg-3′ and 5′-ggggcttggtatatatgtgg-3′; catalase: 5′-tcgagtggccaactaccagcgtg-3′ and 5′-gtacttgtccagaagagcctggatg-3′; GPX1: 5′-aagagattctgaattccctcaagtacg-3′ and 5′-accaggaacttctcaaagttccagg-3′; HO1: 5′-acagcatgccccaggatttgtc-3′ and 5′-agaaggccaggtcctgctccagggcag-3′;GAPDH: 5′-cgaccactttgtcaagctca-3′ and 5′-aggggtctacatggcaactg-3′. Specificity of amplified PCR fragments was confirmed by DNA sequence analysis.

### Quantitative PCR

Quantitative PCR was carried out using an ABI Prism 7000 sequence detection system with standard temperature protocol and QuantiTect SYBR Green PCR Master Mix reagent (Qiagen) in triplicates. 300 nM concentrations of the following primer pairs were used for the reactions: HSP70 (human): forward, 5′-atcatcagcggactgtaccag-3′; reverse, 5′-ctaatctacctcctcaatggtg-3′ HSP70 (mouse): forward, 5′- acaagtgccaggaggtcatctc-3′; reverse, 5′- tctaatccacctcctcgatggtg-3′and GAPDH: forward, 5′-cgaccactttgtcaagctca-3′ and reverse, 5′-aggggtctacatggcaactg-3′. All amplifications were carried out in MicroAmp optical 96-well reaction plates with optical adhesive covers (Applied Biosystems).

### Small RNA Interference

The 21-nt duplex small interfering (si) RNA pools for HSF1 (Stealth RNAi), and control siRNAs (random; 5′-NNACTCTATCTGCACGCTGAC-3′) were purchased from Invitrogen. Cells (5×10^5^ cells/well in a 12-well plate) were incubated for 24 h, and transfected either with HSF1 siRNA or control random siRNA (siRandom) duplexes (80 nmoles each) using Lipofectamine RNAiMAX (Invitrogen). After 2 to 3 days, cells were used for analysis for western blots and cell viability. Transfection efficiency (usually >50%) was assessed in parallel wells by transfection with pEGFP expression vector (BD Biosciences Clontech, Mountain View, CA).

### HSF1 Overexpression

HSF1-/- MEF cells were transfected with HA-HSF1 expression vectors, the wild-type HSF1 (pHA-HSF1 wild type) and the mutant HSF1 carrying S121A (pHA-HSF1S121A), using the Lipofectamine LTX Transfection Reagent (Invitrogen). Cells were harvested 2 to 3 days after transfection for western blots or RT-PCR.

### Folding Recovery Assay

Cells were transiently transfected with a pRL-TK reporter plasmid (Promega Corp., Madison, WI) and luciferase reporter plasmids containing GRE using the Lipofectamine LTX Transfection Reagent. After full activation of GER-mediated transcription by 10 mM dexamethasone for 10 to 14 h, transfectants were washed and exposed to heat at the indicated temperature with or without 0.25 to 0.5 mM H_2_O_2_. After heat exposure, protein refolding activity was evaluated by measurement of luciferase activity using a luminometer (Mini Lumat LB 9506) and normalized to *Renilla* luciferase activity. To detect the effect of HSF1 on folding activity, either the pHA-HSF1 wild type?or its vehicle was co-transfected with the reporter plasmids. Alternatively, A172 cells were transfected with either siHSF1 or siRandom prior to GRE transfection.

### Chromatin Immunoprecipitation Analysis

The protein-DNA interaction was evaluated using the ChIP assay kit (Upstate Biotechnical) according to the manufacture's protocol. Cells were treated as indicated and fixed with formaldehyde for 10 min at 37°c. The DNA-protein complex was immunoprecipitated using anti-HSF1 or unimmunized rabbit serum (1 µg) antibody overnight at 4°c and evaluated by PCR amplification using specific primers, i.e., primers for HSP70 promoter, 5′-gaagactctggagagttctg-3′ and 5′-ccctgggcttttataagtcg-3′. Sensitivity of PCR amplification and sample quality were evaluated on the recovered probe DNA after fixation, sonication and nuclear extract (input fraction). Three independent experiments were performed and similar results were obtained.

### Statistical Analysis

Statistical analysis was evaluated using Student's *t* test (SPSS® program version 10.1; San Rafael, CA). P<0.05 was considered statistically significant.

## References

[1] Finkel T, Holbrook NJ (2000). Oxidants, oxidative stress and the biology of ageing.. Nature.

[2] Klein JA, Ackerman SL (2003). Oxidative stress, cell cycle, and neurodegeneration.. J Clin Invest.

[3] Morimoto RI (1998). Regulation of the heat shock transcriptional response: cross talk between a family of heat shock factors, molecular chaperones, and negative regulators.. Genes Dev.

[4] Sang-Gun Ahn, Thiele DennisJ (2003). Redox regulation of mammalian heat shock factor 1 is essential for Hsp gene activation and protection from stress.. Genes Dev.

[5] McMillan DR, Xiao X, Shao L, Graves K, Benjamin IJ (1998). Targeted disruption of heat shock transcription factor 1 abolishes thermotolerance and protection against heat-inducible apoptosis.. J Biol Chem.

[6] Zhang Y, Huang L, Zhang J, Moskophidis D, Mivechi NF (2002). Targeted disruption of hsf1 leads to lack of thermotolerance and defines tissue-specific regulation for stress-inducible Hsp molecular chaperones.. J Cell Biochem.

[7] Chan WH, Yu JS (2000). Inhibition of UV irradiation-induced oxidative stress and apoptotic biochemical changes in human epidermal carcinoma A431 cells by genistein.. J Cell Biochem.

[8] Valenzuela MT, Núñez MI, Villalobos M, Siles E, McMillan TJ (1997). A comparison of p53 and p16 expression in human tumor cells treated with hyperthermia or ionizing radiation.. Int J Cancer.

[9] Fernandez J, Yaman I, Sarnow P, Snider MD, Hatzoglou M (2002). Regulation of internal ribosomal entry site-mediated translation by phosphorylation of the translation initiation factor eIF2alpha.. J Biol Chem.

[10] Inouye S, Izu H, Takaki E, Suzuki H, Shirai M (2004). Impaired IgG production in mice deficient for heat shock transcription factor 1.. J Biol Chem.

[11] Ito H, Shimojo T, Fujisaki H, Tamamori M, Ishiyama S (1999). Thermal preconditioning protects rat cardiac muscle cells from doxorubicin-induced apoptosis.. Life Sci.

[12] Wang X, Khaleque MA, Zhao MJ, Zhong R, Gaestel M (2006). Phosphorylation of HSF1 by MAPK-activated protein kinase 2 on serine 121, inhibits transcriptional activity and promotes HSP90 binding.. J Biol Chem.

[13] Burdon RH, Gill VM, Rice-Evans C (1987). Oxidative stress and heat shock protein induction in human cells.. Free Radic Res Commun.

[14] Skibba JL, Powers RH, Stadnicka A, Cullinane DW, Almagro UA (1991). Oxidative stress as a precursor to the irreversible hepatocellular injury caused by hyperthermia.. Int J Hyperthermia.

[15] McAnulty SR, McAnulty L, Pascoe DD, Gropper SS, Keith RE (2005). Hyperthermia increases exercise-induced oxidative stress.. Int J Sports Med.

[16] Heine GF, Horwitz AA, Parvin JD (2008). Multiple Mechanisms Contribute to Inhibit Transcription in Response to DNA Damage.. J Biol Chem.

[17] Ranu RS, London IM (1976). Regulation of protein synthesis in rabbit reticulocyte lysates: purification and initial characterization of the cyclic 3′:5′-AMP independent protein kinase of the heme-regulated translational inhibitor.. Proc Natl Acad Sci U S A.

[18] Harding HP, Zhang Y, Bertolotti A, Zeng H, Ron D (2000). Perk is essential for translational regulation and cell survival during the unfolded protein response.. Mol Cell.

[19] Yoshida H, Matsui T, Yamamoto A, Okada T, Mori K (2001). XBP1 mRNA is induced by ATF6 and spliced by IRE1 in response to ER stress to produce a highly active transcription factor.. Cell.

[20] Kalmar B, Greensmith L (2009). Induction of heat shock proteins for protection against oxidative stress.. Adv Drug Deliv Rev.

[21] Cullinan SB, Zhang D, Hannink M, Arvisais E, Kaufman RJ (2003). Nrf2 is a direct PERK substrate and effector of PERK-dependent cell survival.. Mol Cell Biol.

[22] Liu Y, Adachi M, Zhao S, Hareyama M, Koong AC (2009). Preventing oxidative stress: a new role for XBP1.. Cell Death Differ.

[23] Sucholeiki R (2005). Heatstroke.. Semin Neurol.

[24] Jardine DS (2007). Heat illness and heat stroke.. Pediatr Rev.

[25] Zhang Y, Adachi M, Kawamura R, Imai K (2006). Bmf is a possible mediator in histone deacetylase inhibitors FK228 and CBHA-induced apoptosis.. Cell Death Differ.

